# Single-cell profiling and functional screening reveal crucial roles for lncRNAs in the epidermal re-epithelialization of human acute wounds

**DOI:** 10.3389/fsurg.2024.1349135

**Published:** 2024-02-26

**Authors:** Yunting Xiao, Chenyang Zhang, Xiuping Liu, Yong Yang, Ning Xu Landén, Zhao Zhang, Dongqing Li

**Affiliations:** ^1^ Hospital for Skin Diseases, Institute of Dermatology, Chinese Academy of Medical Sciences and Peking Union Medical College, Nanjing, China; ^2^ Key Laboratory of Basic and Translational Research on Immune-Mediated Skin Diseases, Chinese Academy of Medical Sciences, Nanjing, China; ^3^ Jiangsu Key Laboratory of Molecular Biology for Skin Diseases and STIs, Nanjing, China; ^4^ MOE Key Laboratory of Metabolism and Molecular Medicine, Department of Biochemistry and Molecular Biology, School of Basic Medical Sciences, Fudan University, Shanghai, China; ^5^ Department of Pathology, School of Basic Medical Sciences, Fudan University, Shanghai, China; ^6^ Dermatology and Venereology Division, Department of Medicine Solna, Center for Molecular Medicine, Karolinska Institutet, Stockholm, Sweden

**Keywords:** single-cell sequencing, long noncoding RNA, wound healing, re-epithelialization, keratinocyte differentiation

## Abstract

**Objectives:**

Re-epithelialization is an important physiological process for repairing skin barrier function during wound healing. It is primarily mediated by coordinated migration, proliferation, and differentiation of keratinocytes. Long noncoding RNAs (lncRNAs) are essential components of the noncoding genome and participate in various biological processes; however, their expression profiles and function in re-epithelialization during wound healing have not been established.

**Methods:**

We investigated the distribution of lncRNAs during wound re-epithelialization by comparing the genomic profiles of uninjured skin and acute wound (AW) from healthy donors. We performed functional screening of differentially expressed lncRNAs to identify the important lncRNAs for re-epithelialization.

**Results:**

The expression of multiple lncRNAs is changed during human wound re-epithelialization process. We identified VIM-AS1, SMAD5-AS1, and LINC02581 as critical regulators involved in keratinocyte migration, proliferation, and differentiation, respectively.

**Conclusion:**

LncRNAs play crucial regulatory roles in wound re-epithelialization. We established lncRNA expression profile in human acute wounds compared with intact skin, offering valuable insights into the physiological mechanisms underlying wound healing and potential therapeutic targets.

## 1 Introduction

Wound healing is a fundamental and complex biological process that maintains skin integrity. Re-epithelialization is an important process for restoring skin barrier function during wound healing, which is primarily mediated by coordinated migration, proliferation, and differentiation of keratinocytes at the wound edge. Following skin injury, keratinocytes are activated by damage-associated molecular patterns (DAMPs) and secrete multiple proinflammatory cytokines and growth factors (1). These factors further recruit neutrophils, monocytes, and macrophages to the injury site, which amplify the inflammatory response. Upon activation, keratinocytes alter the cytoskeleton network, and cell–cell interactions are disrupted by PKCα signaling and switching from α6β4 to α3β1 integrin for laminin-5 (LN5) binding to release keratinocytes from the original site (2). At the wound edge tongue, keratinocytes also proliferate to produce adequate supply of cells to cover the wound. Growth factors play a major role in the proliferation of keratinocytes, including heparin-binding EGF-like growth factor (HB-EGF), epidermal growth factor (EGF), keratinocyte growth factor (KGF), insulin-like growth factor (IGF)-1, and fibroblast growth factors (FGFs) (3). The neo-epithelium finally commits to a terminal differentiation program to regenerate a stratified epithelium, forming the epidermal barrier (4). Failure in epithelialization contributes to various chronic nonhealing wounds, such as venous ulcer (VU) and diabetic foot ulcer (DFU). Chronic wounds affect approximately 2% of the population, imposing an annual cost of USD 20 billion and presenting a substantial and rising health and economic burden (5). Because of the complex pathophysiology of chronic wounds, few targeted therapeutic drugs have been developed.

The majority of transcripts in the human genome are ultimately processed into nonprotein coding RNA. Long noncoding RNAs (lncRNA) are a large and diverse class, which are defined as transcripts contain more than 200 nucleotides. The number of lncRNA genes in humans ranges from less than 20,000 to over 100,000 and is categorized into five subgroups: intergenic, antisense, sense, intronic, and bidirectional. Many lncRNAs exhibit high tissue- and cell-specific expression patterns, but they are less conserved across species compared with mRNA sequences encoding proteins (6). Nevertheless, the function and biological relevance of the majority of lncRNA have not been annotated. Several lncRNAs play crucial roles in skin wound healing. For example, WAKMAR1 and WAKMAR2 are two injury-regulated lncRNAs that are deeply involved in re-epithelialization and inflammatory response (7). LncRNA-H19 increases dermal fibroblast proliferation and macrophage infiltration to accelerate wound healing in mice (8). LncFAO inhibits the activation of macrophages in the inflammatory phase of wound healing by promoting fatty acid oxidation (FAO) (9). However, the expression of lncRNAs during the wound healing process, particularly during re-epithelialization, has not been systematically analyzed.

We previously used Smart-Seq2 single-cell RNA sequencing to analyze the cell composition and gene expression patterns of epidermal cells of human acute wound (10). In this study, we used this full-length, single-cell transcriptome data to identify the landscape of lncRNAs during re-epithelialization of the wound. Functional screening revealed multiple differentially expressed lncRNAs that were crucial for the migration, proliferation, and differentiation of keratinocytes.

## 2 Results

### 2.1 Detection of lncRNAs and mRNAs in epidermal cells from the skin and acute wound

**Figure 1:**
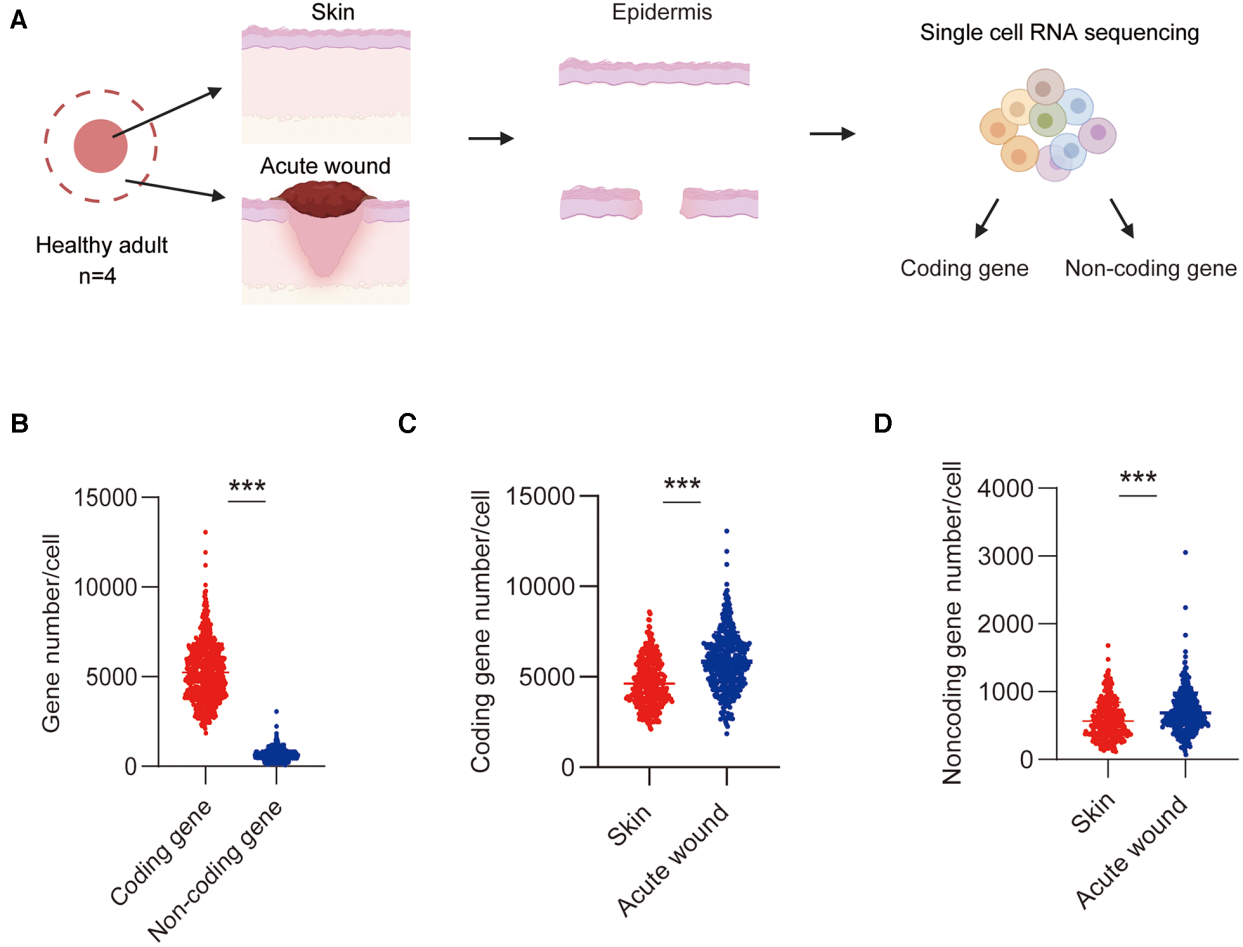
Detection of lncRNAs and mRNAs in epidermal cells in skin and wound. (**A**) Experimental workflow of scRNA-Seq: Single-cell suspensions were dissociated from the epidermis of skin and AW. (**B**) The average number of coding genes and lncRNA genes per cell in skin and wound was detected by scRNA-Seq. (**C**) The average number of coding genes per cell in skin and wound. (**D**) The average number of noncoding genes per cell in skin and wound.

We recently performed a single-cell transcriptome sequencing to characterize the cell composition and gene expression changes that occur during wound healing in humans (10). In this study, we examined the physiological roles of lncRNAs in re-epithelialization. To precisely examine the expression patterns and functions of lncRNAs under physiological conditions, we specifically chose epidermal cells from uninjured skin and Day-7 acute wounds (AW) for analysis in this study. We first investigated the expression patterns of lncRNAs and mRNAs by systematic bioinformatics analyses (Figure 1A). On an average, 3,225 coding genes and 495 noncoding genes in the skin and wound cells were detected as fragments per kilobase of transcript sequence per million base pairs mapped (FPKM) ≥1 in the skin and wound cells (Figure 1B). The number of both coding and noncoding genes in the wound was significantly increased compared with the intact skin, indicating active transcription in the wound environment (Figures 1C,D).

### 2.2 Cell type clustering by using lncRNAs in human acute wound

**Figure 2:**
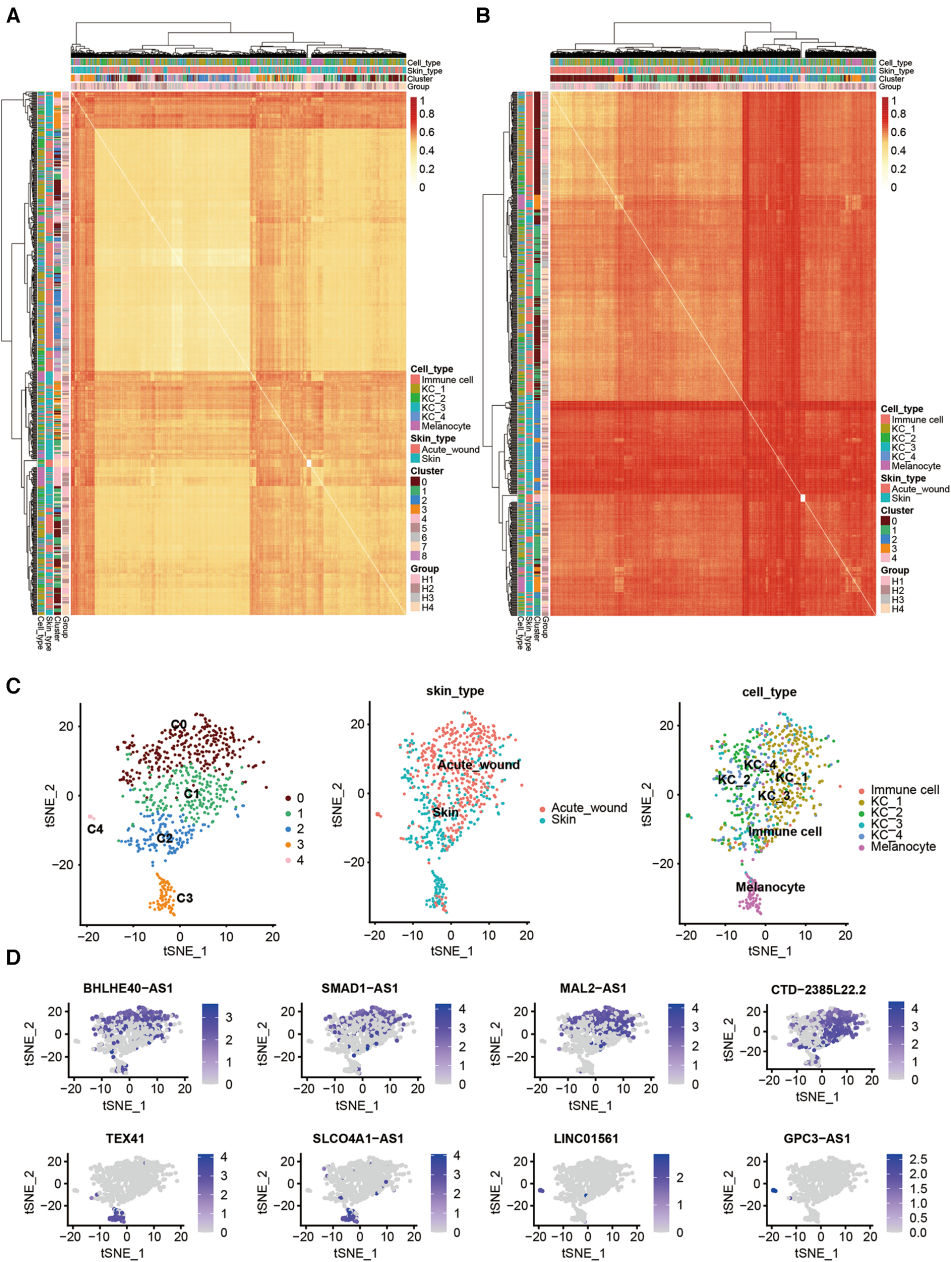
Cell type clustering using lncRNAs and differentially expressed lncRNAs in human acute wound. Heatmap of all single cells (*n* = 707 single cells) analyzed by RNA-Seq with single cells clustered by spatiotemporal location and correlation distance. (**A**) Coding gene; (**B**) lncRNA genes. (**C**) t-Distributed Stochastic Neighbor Embedding (tSNE) plot for clusters marked by lncRNA genes (left), skin types (middle), and cell types (right). (**D**) tSNE plot for the expression patterns of partial marker lncRNA genes in all cells.

To examine the expression of lncRNAs during re-epithelialization, we compared the cell-to-cell distance of coding and noncoding RNA (Figures 2A,B). We found that the expression distance of noncoding RNA was longer than that of coding genes across cells, which suggests that cell clusters analyzed by noncoding RNA are more distinguished than mRNA.

Based on the resulting lncRNA expression profiles, principal component analysis (PCA) successfully clustered epidermal cells into two main cell types: keratinocytes (clusters 0–3) and melanocytes (cluster 4), which is consistent with our previous study. Keratinocytes were further subclustered into four subsets. Interestingly, we identified one KC subset (C4) that was predominant in acute wound (Figure 2C). We further calculated the top differentially expressed lncRNAs for each cell cluster as marker genes, i.e., BHLHE40-AS1, SMAD1-AS1, and MAL2-AS1 for KC_1; CTD-2385l22.2 for KC_2; TEX4 and SLCO4A1-AS1 for KC_3; LINC01561 and GPC3-AS1 for KC_4 (Figure 2D).

**Figure 3:**
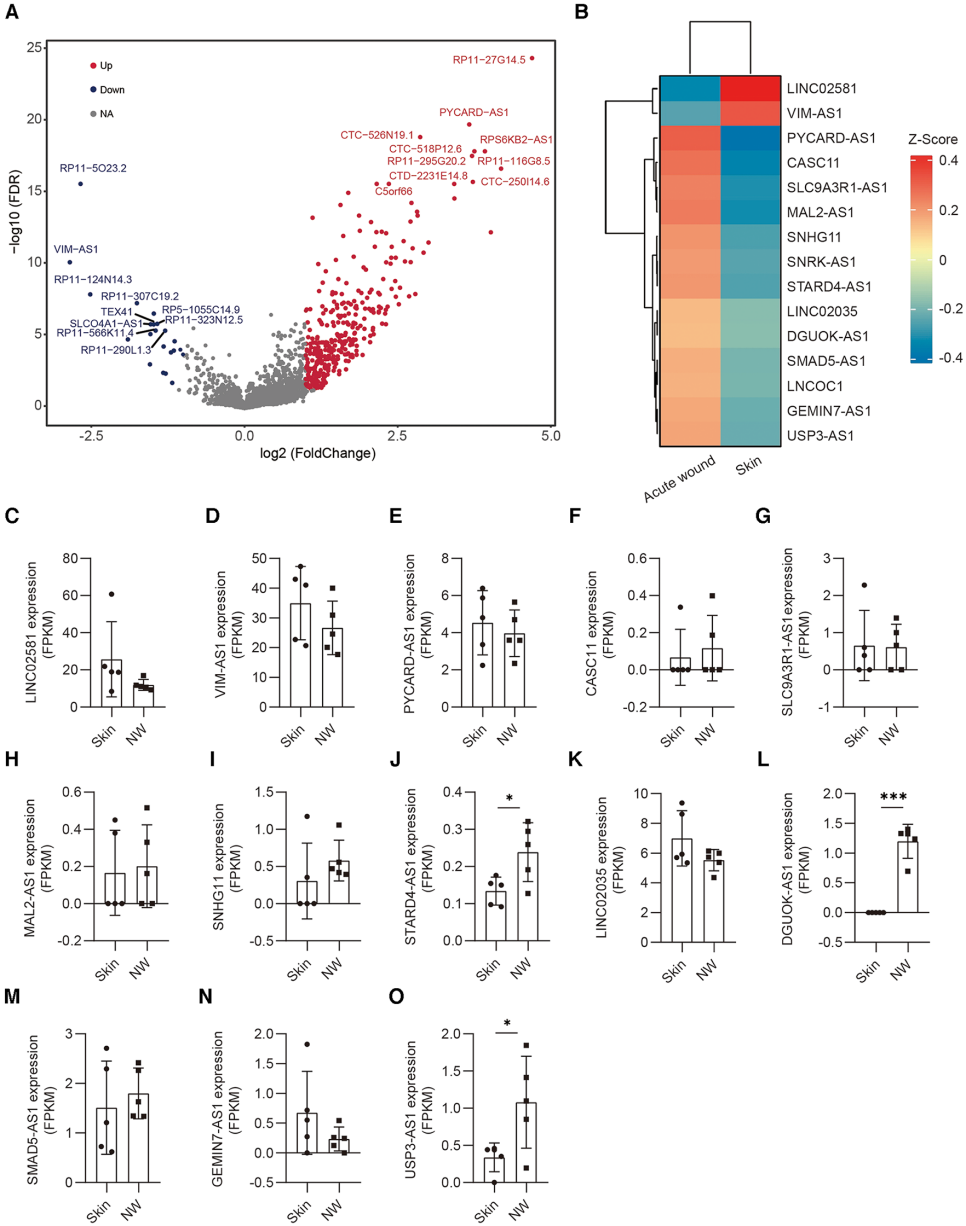
Differentially expressed lncRNAs in the epidermis of acute wound. (**A**) Differentially expressed lncRNAs analyzed by volcano plots between skin and AW. The genes with a log2(FC) ≥1 and an FDR <0.05 are labeled in red, log2(FC) ≤−1 and an FDR <0.05 are labeled in blue. (**B**) Heatmap illustrates the top 15 differentially expressed lncRNAs in acute wound. The expression of LINC02581 (**C**), VIM-AS1 (**D**), PYCARD-AS1 (**E**), CASC11 (**F**), SLC9A3R1-AS1 (**G**), MAL2-AS1 (**H**), SNHG11 (**I**), STARD4-AS1 (**J**), LINC02035 (**K**), DGUOK-AS1 (**L**), SMAD5-AS1 (**M**), GEMIN7-AS1 (**N**), USP3-AS1 (**O**) in the epidermal cells of skin and wound edge.

Next, we performed pairwise whole transcriptome comparisons by using Venice (11). A total of 254 lncRNAs were differentially expressed in the keratinocytes of human acute wound, including 218 upregulated and 36 downregulated lncRNAs [Log10(FDR) >3, Log2[fold change (FC)] >0.5] (Figure 3A). The top 15 lncRNAs exhibiting the greatest expression difference between skin and AW were selected, of which two was downregulated, whereas 13 were upregulated in AW (Figure 3B). We next analyzed the conservation of these 15 lncRNAs across species. Although most of these lncRNAs are not conserved in zebrafish, we were able to identify the homologous genes of 6 lncRNAs (Table S1). Among the differentially expressed genes, multiple lncRNAs were previously found to regulate cell proliferation and migration, including VIM-AS1 (12, 13), CASC11 (14, 15), DGUOK-AS1 (16, 17), SNHG11 (18, 19), and SMAD5-AS1 (20).

To further confirm the expression pattern of these selected lncRNAs, we conducted additional validation of lncRNA expression using bulk RNA sequencing data obtained from epidermal cells in human skin and wound edges. Our analysis revealed a consistent trend in lncRNA expression between bulk sequencing and single-cell RNA sequencing datasets (Figures 3C–O). Notably, STARD4-AS1 (Figure 3J), DGUOK-AS1(Figure 3L), and UPS3-AS1(Figure 3O) exhibited a significant upregulation in acute wounds, aligning with observations from single-cell RNA sequencing data. Conversely, LINC02581 (Figure 3A) and VIM-AS1(Figure 3B) demonstrated decreased expression trend in acute wounds (Figure 3B).

### 2.3 Functional screening of lncRNAs regulating migration and proliferation of keratinocyte progenitor cells

**Figure 4:**
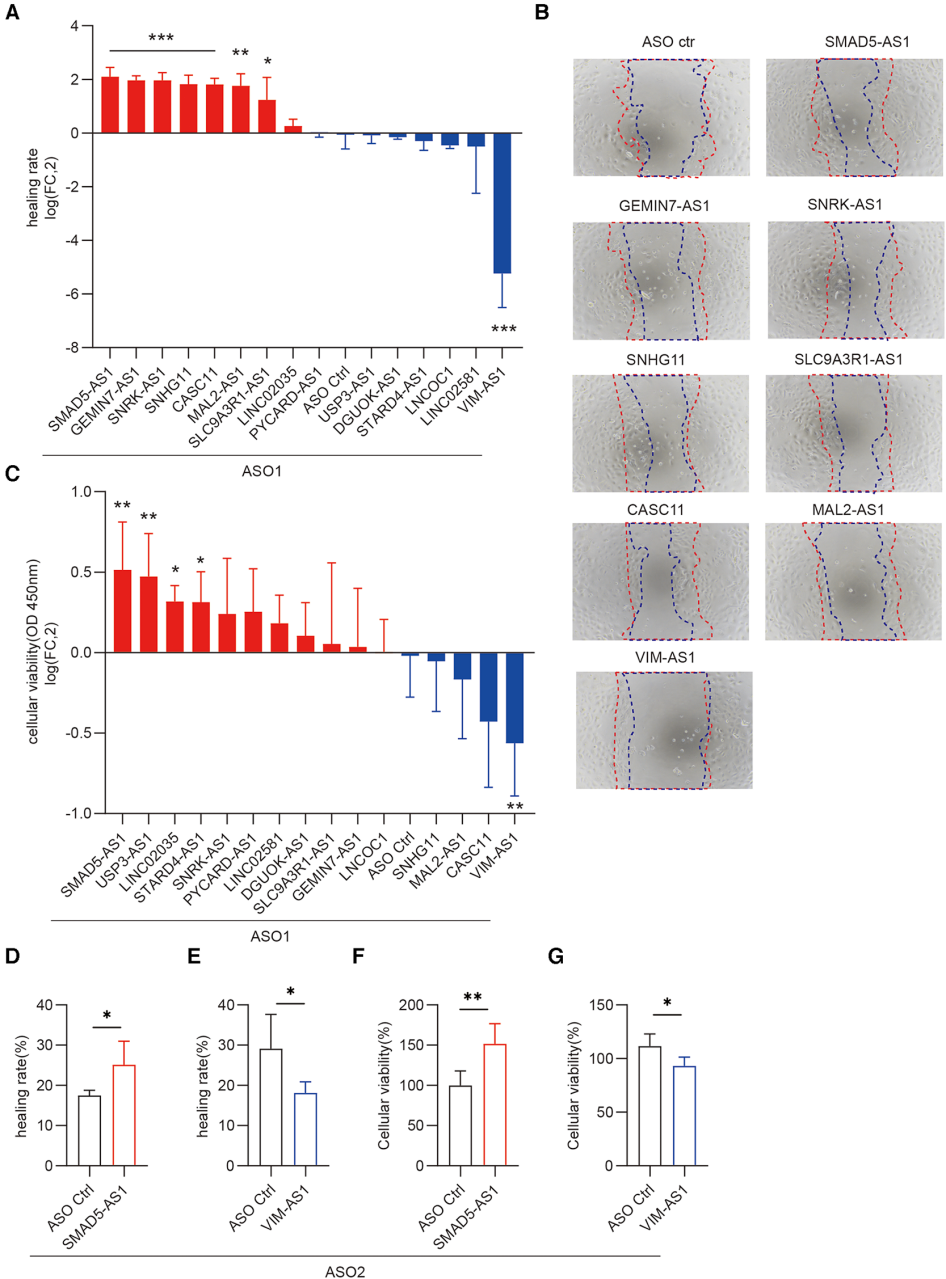
Functional screening of lncRNAs regulating migration and proliferation of keratinocyte progenitor cells. Scratch wound assay of keratinocytes transfected with 20 nM ASOs targeting these lncRNAs and ASO control for 24 h (*n* = 4). Photographs were taken at 0 and 24 h after the scratch. Healing rates were quantified by measuring the area of the scratched region (**A**) Representative photographs of migrating keratinocytes at the indicated timepoint (**B**) The data are represented as the mean ± SD of four independent experiments (*n* = 4, *p < 0.05, **p < 0.01, and ***p < 0.001). (**C**) CCK8 assay was used to detect the viability of keratinocytes to knockdown these lncRNAs (n = 6). Cellular viability = (mean absorbance of experimental group/mean absorbance of control group) ×100%. (**D,E**) Quantification of the healing rate was performed after knockdown of SMAD5-AS1 and VIM-AS1 using the second ASO. (**F,G**) CCK-8 cell proliferation assay was conducted to assess the viability of keratinocytes following the knockdown of SMAD5-AS1 and VIM-AS1 using the second ASO.

Migration and proliferation of keratinocytes are required for re-epithelialization during wound healing. To determine the potential impact of candidate lncRNAs on the proliferation and migration of human keratinocyte progenitor cells, we transfected antisense oligonucleotides (ASOs) targeting each lncRNA, followed by cell scratch assay or cell proliferation assay. We found that silencing SMAD5-AS1, GEMIN7-AS1, SNRK-AS1, SNHG11, CASC11, MAL2-AS1, and SLC9A3R1-AS1 increased keratinocyte migration, whereas silencing VIM-AS1 decreased migration (Figures 4A,B). Moreover, the downregulation of SMAD5-AS1, USP3-AS1, LINC02035, and STARD4-AS1 enhanced the proliferation of keratinocytes, whereas the knockdown of VIM-AS1 inhibited keratinocyte viability (Figure 4C). Among these 16 lncRNAs, silencing SMAD5-AS1 and VIM-AS1 had the greatest impact on the proliferation and migration of keratinocytes. To validate these findings, we conducted additional experiments by synthesizing another ASOs that targeted distinct loci of SMAD5-AS1 and VIM-AS1. The depletion of SMAD5-AS1 using ASO2 markedly enhanced the migration and proliferation of keratinocytes (Figures 4D,F). Conversely, the reduction of VIM-AS1 through ASO2 significantly inhibited keratinocytes’ migration and proliferation (Figures 4E,G).

### 2.4 Functional screening of lncRNAs regulating differentiation of keratinocyte progenitor cells

**Figure 5:**
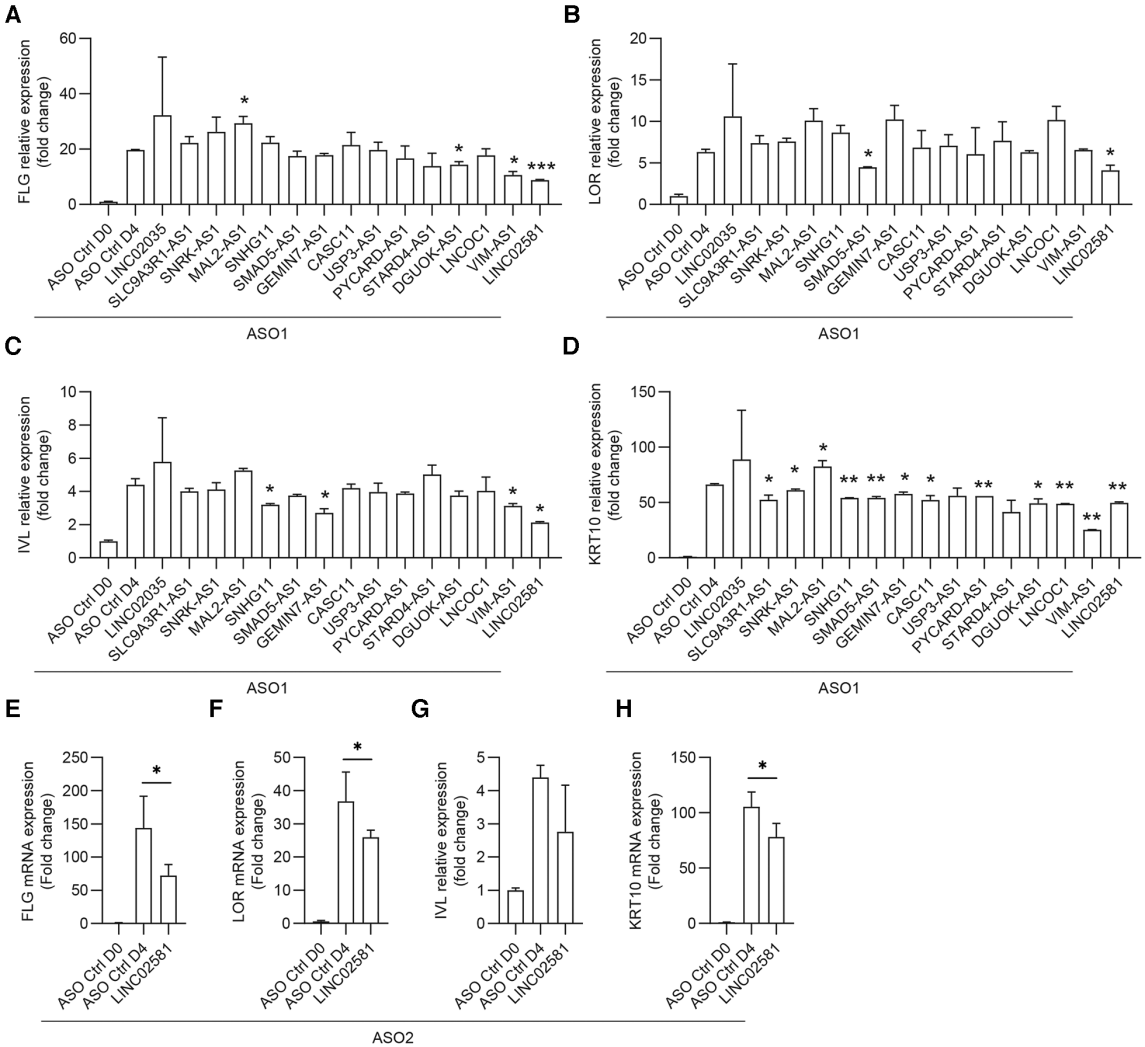
Functional screening of lncRNAs regulating differentiation of keratinocyte progenitor cells. The human primary keratinocytes were transfected with 20 nM ASOs targeting the lncRNAs or ASO controls for 24 h followed by induction of differentiation with 1.2 mM CaCl_2_. The expression of FLG (**A**), LOR (**B**), IVL (**C**), and KRT10 (**D**) were measured by qRT-PCR. (**E–H**) The expression of FLG (**E**), LOR (**F**), IVL (**G**), and KRT10 (**H**) were measured by qRT-PCR after LINC02581 knockdown by using the second ASO.

In the wound-edge epithelium, migrating and proliferating keratinocytes are primarily undifferentiated cells. After keratinocyte progenitor cells cover the wound, timely terminal differentiation is necessary to restore epidermal barrier function. To determine the function of these selected lncRNAs in the differentiation of keratinocyte progenitor cells, we transfected ASOs for these lncRNAs into progenitor cells. Once the cells were confluent, we treated the cells with 1.2 mM CaCl_2_, which is widely used to induce keratinocyte differentiation (21). The results indicated that silencing MAL2-AS1 promoted CaCl_2_-induced filaggrin (FLG) expression, whereas silencing DGUOK-AS1, VIM-AS1, and LINC02581 had the opposite effect (Figure 5A). We found that silencing SNHG11, GEMIN7-AS1, VIM-AS1, and LINC02581 inhibited CaCl_2_-induced involucrin (IVL), whereas silencing SMAD5-AS1, LINC02581 inhibited CaCl_2_-induced loricrin (LOR) (Figures 5B,C). IVL and LOR were another terminal differentiation marker genes of keratinocytes. We also examined the expression of the early differentiation marker gene keratin 10 (KRT10) and observed a similar expression pattern after MAL2-AS1, SMAD5-AS1, VIM-AS1, GEMIN7-AS1, DGUOK-AS1, and LINC02581 knockdown (Figure 5D). In consistent with this, the second ASO which targeted different loci of LINC02581 also decreased the expression of these early and late differentiation marker genes (Figures 5E–H).

### 2.5 Correlation analysis of lncRNA and protein-coding genes

**Figure 6:**
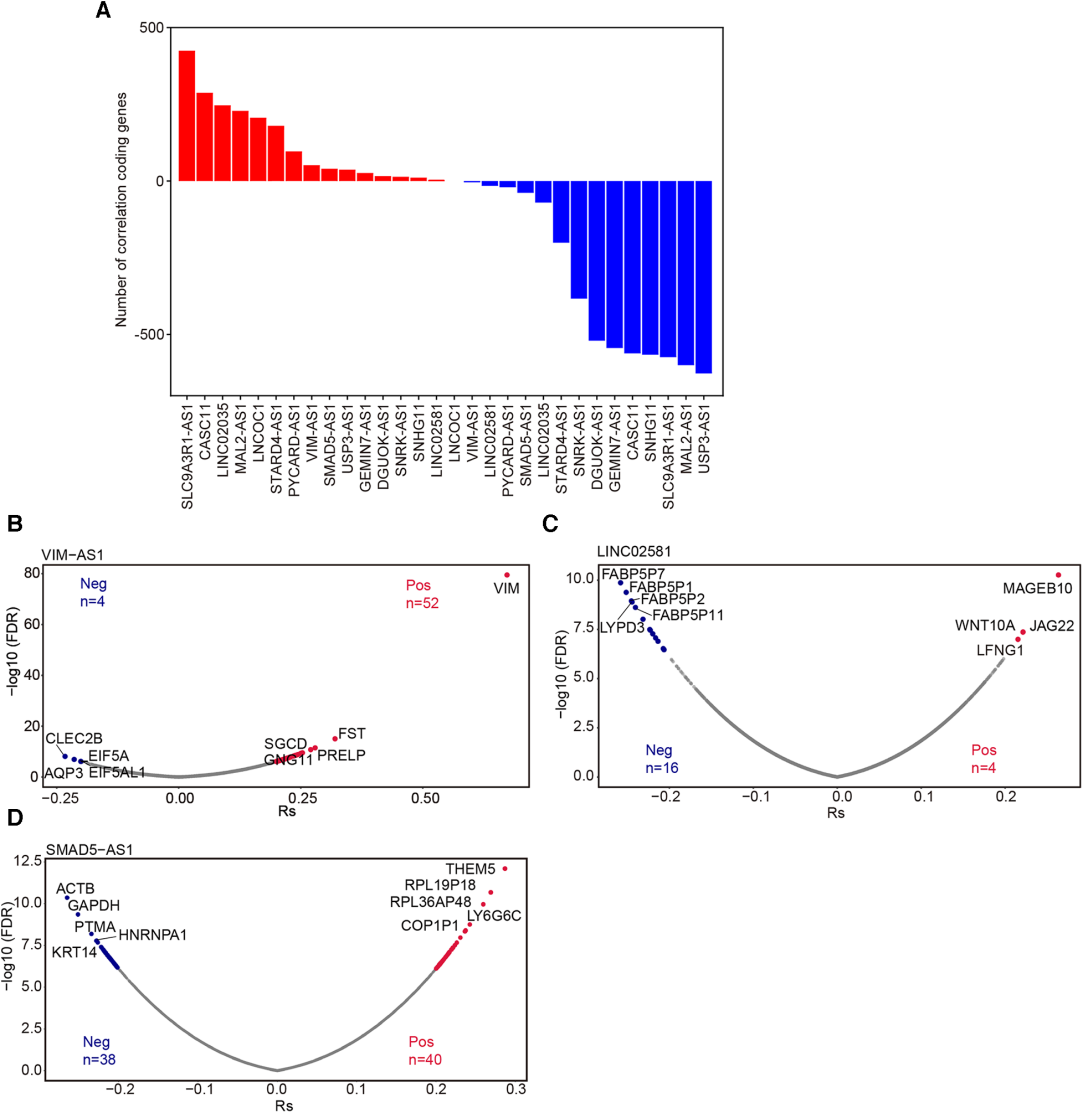
Correlation analysis of lncRNA and protein-coding genes. (**A**) The number of coding genes associated with these 15 lncRNAs. Volcano plot showed the mRNAs significantly associated withVIM-AS1 (**B**), LINC02581(**C**) and SMAD5-AS1(**D**) (FDR <0.05 and |RS|≥ 0.2). Pos: Positive correlation mRNAs; Neg: Negative correlation mRNA.

LncRNAs modulate gene expression either in cis (affecting neighboring genes) or in trans (influencing distant genes), and their correlation with the expression of target transcripts can be either negative or positive (22). To explore the coding genes correlated with these lncRNAs, we conducted Spearman correlation analysis (23). The number of correlated coding RNAs varied substantially for each lncRNA (FDR <0.05 and |Rs| ≥0.2) (Figure 6A, Supplementary Figure S1A–L). Specifically, our analysis identified 56, 78, and 20 coding RNAs closely correlated with VIM-AS1, LINC02581, and SMAD5-AS, respectively (Figures 6B–D).

Moreover, we categorized these mRNAs into groups showing positive or negative correlation with each respective lncRNA for subsequent EnrichR pathway analysis (24). The results revealed that the coding RNAs positively correlated with PYCARD-AS1 were associated with ATP synthesis and respiratory electron transport (Supplementary Figure S2A). The positively correlated coding RNAs of STARD4-AS1 were linked to the cell cycle (Supplementary Figure S2B), while those of MAL2-AS1 were involved in fatty acid metabolism (Supplementary Figure S2C), and the positively correlated coding RNAs of CASC11 were associated with mTOR signaling (Supplementary Figure S2D). These findings suggest potential regulatory roles for PYCARD-AS1, STARD4-AS1, MAL2-AS1, and CASC11 in these signaling pathways. Unfortunately, we did not observe significantly enriched functions for other lncRNAs.

## 3 Discussion

Wound healing is a complex, but precisely regulated process that maintains skin integrity. After skin injury, epidermal cells are activated and subsequently proliferate and migrate into the wound bed to create a new barrier between the wound and environment. LncRNAs are important regulators in many physiological processes, including development, adipogenesis, and circadian rhythm (25). However, the role of lncRNAs in wound healing remains largely unexplored. In this study, we used systemic bioinformatic analysis to establish lncRNA transcriptional profiles of the epidermal cells in human acute wounds. Combined with functional screening, we identified multiple lncRNAs that are crucial for keratinocyte progenitor cell proliferation and migration, including VIM-AS1 and SMAD5-AS1. We also identified MAL2-AS1 and LINC02581, which contribute to the differentiation of keratinocyte progenitor cells. Since the current commercial single-cell sequencing technology only captures polyadenylated transcripts, further scRNA-seq, which can detect non-polyadenylated transcripts (mainly noncoding RNA), may reveal a comprehensive expression profile of noncoding RNAs involved in wound healing.

Wound healing is a hierarchically orchestrated process comprising multiple cell types and factors. Given the cell type and tissue specificity of lncRNA, it is necessary to identify the cell type-specific lncRNAs in wound (26). Bulk RNA sequencing measures average expression levels across cell populations, whereas scRNA-seq enables to detecting gene expression in individual cells. Although mice are most commonly used in preclinical studies, human and mice wound are distinct. Human wound primarily heal through re-epithelialization, whereas rodents primarily heal by contraction (27). This intrinsic difference may lead to distinct gene expression patterns in the two organisms. In this study, we identified 254 differentially expressed lncRNAs in human wound epidermal keratinocytes from our previous scRNA-seq data. We found that the expression of most lncRNAs were increased in wound epidermal keratinocytes. This phenomenon demonstrates that lncRNA and mRNA exert different transcriptional regulation mechanisms during wound healing.

We further validated the expression of the top 15 differentially expressed lncRNAs using bulk RNA sequencing data from wound edge epidermal cells, and we observed a consistent trend. However, given the limited number of donors in the bulk RNA sequencing dataset, certain lncRNAs did not exhibit a significant difference in acute wounds. To enhance confidence in these findings, additional investigation with a larger number of donors is required to further confirm the expression patterns of these lncRNAs.

Approximately 30,000–60,000 human lncRNAs have been identified in the human genome. However, the function of most lncRNAs remains unclear (28). Herein, we designed ASOs to target 15 differentially expressed lncRNAs. By employing a small-scale functional screen to analyze the proliferation and migration of keratinocyte progenitors following lncRNA knockdown, we found that VIM-AS1 promoted the migration of keratinocytes, whereas SMAD5-AS1, GEMIN7-AS1, SNRK-AS1, SNHG11, CASC11, MAL2-AS1, and SLC9A3R1-AS1 inhibited keratinocyte migration. In addition, VIM-AS1 promoted keratinocyte proliferation, whereas SMAD5-AS1, USP3-AS1, LINC02035, and STARD4-AS1 mediated the opposite effect. Therefore, we propose that the deficiency of VIM-AS1, SMAD5-AS1, and LINC02581 may contribute to the pathogenesis of chronic wounds. LncRNAs frequently function by interacting with various molecular targets, contributing to diverse cellular processes. Conducting transcriptome studies following specific lncRNA manipulation is essential to analyze potential impacts on other biological processes. Additionally, *in vivo* experiments are necessary to further validate the functions attributed to these identified lncRNAs. To date, only a few lncRNAs have been characterized in wound healing. The lncRNAs screened in this study may represent new therapeutic targets for chronic wound.

VIM-AS1 is an antisense RNA for vimentin and is located on chr10: 17,214,239–17,229,985. Multiple studies have indicated that VIM-AS1 promotes cell proliferation, migration, and epithelial–mesenchymal transition in human cancers (12, 13). In diabetes, Omidvar et al. showed that decreased VIM-AS1 levels in PBMCs are associated with diabetes in the Iranian population (29). In diabetic retinopathy patients, VIM-AS1 is downregulated in plasma samples and may inhibit high glucose-induced apoptosis of retinal pigment epithelial cells through miR-29 (30). These studies are consistent with our results, which revealed that VIM-AS1 knockdown significantly decreased the proliferation and migration of keratinocyte progenitors. Antisense lncRNA can stimulate or inhibit the expression of sense protein-coding or noncoding genes through different mechanisms. Thus, further studies are warranted to determine whether VIM-AS1 regulates vimentin expression to promote the proliferation and migration of keratinocytes. Given the complexity of the wound environment, it is crucial to identify the factors that regulate VIM-AS1 expression in wound.

SMAD5-AS1 is another antisense lncRNA which expression is significantly upregulated in the keratinocyte of wound. Previous studies revealed that SMAD5-AS1 promotes the epithelial–mesenchymal transition in nasopharyngeal carcinoma by sponging miRNA-106a-5p or miRNA-195 to upregulate SMAD5 expression (31). However, in diffuse large B cell lymphoma, SMAD5-AS1 inhibits tumor cell proliferation by sponging miR-135b-5p to increase the expression of the adenomatous polyposis coli (APC) genes (20). The opposite effect of SMAD5-AS1 in tumor cells suggests that this lncRNA plays distinct roles in different tissues. In our study, we found that SMAD5-AS1 knockdown significantly increased the proliferation and migration of keratinocyte progenitor cells. However, the mechanism for the regulation of keratinocyte function by SMAD5-AS1 warrants further investigation.

Previous studies have shown that several lncRNAs can modulate epidermal differentiation. TINCR, uc.291, PRANCR, and HOXC13-AS positively regulate keratinocyte differentiation, whereas ANCR and LINC00941 generate the opposite effect. TINCR is the first lncRNA characterized in human skin, which binds to the STAU1 protein to stabilize mRNAs associated with differentiation. Interestingly, recent studies showed that although TINCR is not a noncoding RNA, it encodes a ubiquitin-like protein that promotes keratinocyte proliferation in wound healing and suppresses tumor growth in squamous cell carcinoma. A recent study predicted that approximately 40% of lncRNAs and pseudogene RNAs expressed in human cells are translated (32). In this study, we found that knockdown of LINC02581 significantly decreased CaCl_2_-induced differentiation genes expression. Our study also revealed that LINC02581 was the most significantly downregulated lncRNA among the small number of decreased lncRNAs in the wound-edge epidermal cells. Interestingly, LINC02581 was recently annotated as the protein-coding gene RNF227. Further investigation is required to confirm the translational capacity of LINC02581 and to elucidate the role of RNF227 in keratinocyte differentiation.

Long non-coding RNAs (lncRNAs) have demonstrated their capacity to regulate gene expression, playing pivotal roles in the control of chromosome structure and function, and influencing various facets of mRNA dynamics, such as splicing, stability, translation, and degradation. These characteristics position lncRNAs as promising contenders for addressing diseases associated with these biological processes. In the context of chronic wounds, the dysregulation of specific lncRNAs, such as WAKMAR1 (33) and H19 (8) in diabetic foot ulcers, WAKMAR2 (7) in venous ulcers, has been implicated in pathogenesis. While none of these lncRNAs have yet entered clinical trials for wound treatment, their substantial clinical therapeutic potential is evident. Noteworthy examples include the delivery of lncH19 (34) and LINC01435 (35) via exosomes and lentivirus-mediated knockdown of lnc-URIDS (36), which demonstrated the promotion of wound healing in diabetic mice.

The translation of research findings from laboratory bench to clinical application for lncRNA-based treatments in chronic wounds involves a multifaceted process. This includes the identification and functional characterization of key lncRNAs, the development of delivery systems, and the optimization of therapeutic approaches, among other steps. Importantly, the translation of lncRNA-based therapies from bench to bedside is an intricate and iterative journey necessitating collaboration between researchers, clinicians, regulatory agencies, and industry partners. Successful implementation of these therapies has the potential to revolutionize the treatment landscape for chronic wounds, providing novel and targeted therapeutic options that could significantly improve patient outcomes.

In summary, this study comprehensively analyzed the expression pattern of lncRNAs in human acute wound. By performing a small-scale functional screening, we identified SMAD5-AS1, VIM-AS1, and LINC02581 as crucial regulators of epidermal re-epithelialization. LncRNAs have garnered increased interest as wound healing targets. Our findings unravel the important roles for lncRNAs in skin tissue repair. Further studies are needed to identify their underlying mechanism and therapeutic potential.

## 4 Materials and methods

### 4.1 Single-cell RNA sequencing analysis

Single-cell RNA sequencing data were collected from a previous study (GSE137897). We used FastQC (37) to filter out low-quality reads and used Hisat2 (38) to align high-quality reads to human genome (hg38). Expression level of each coding and noncoding genes was calculated by StringTie (39) with Fragments per Kilobase Million (FPKM) methods, with annotation collected from UCSC (https://genome.ucsc.edu/). Expression matrixes were further analyzed by Seurat (Version 4.0.5 with default parameters, https://satijalab.org/seurat/). Next, 707 individual cells were used for further investigation. calculating clusters were used Louvain algorithm with 15 principal components, and resolution of 0.7. The t-distributed Stochastic Neighbor Embedding (t-SNE) assay was used to classify each cell into different cell type based on reported markers (10). The expression of genes in individual cells was showed with *FeaturePlot* function in Seurat. We also calculated expression distance of between two cells with Euclidean distance (R, version 4.0.5) by coding genes and noncoding genes, respectively.

### 4.2 Screening for mRNAs correlated with lncRNA

We calculated the interaction between lncRNA and mRNA using spearman's rank correlations and considered |Rs| ≥0.2 and FDR <0.05 as significance. These correlated mRNAs were used to investigate pathway analyses by using the Enrichr website database.

### 4.3 Cell culture and treatment

Normal human epidermal keratinocytes-adults (Lifeline® Cell Technology) were cultured in DermaLife® K basic medium supplemented with DermaLife K LifeFactors (Lifeline® Cell Technology) and 1% penicillin/streptomycin at 37°C in a humidified incubator with 5% CO_2_ (ThermoFisher Scientific). To silence lncRNA expression, keratinocytes from the third passage were transfected when they reached 50%–60% confluence in a 96-well plate. Lipofectamine™ RNAimax (ThermoFisher Scientific) was used to deliver 20 nM ASOs or a control ASO (Genepharma). The calcium concentration was increased to 1.2 mM in the medium to induce cell differentiation. Following ASO transfection, keratinocyte differentiation was induced by adding medium containing 1.2 mM Ca^2+^ for four days.

The list of ASOs are as follows: LINC02581: GTAGAGCACGTTGCTAGGCA, TGAAAGGACGGTAGCAGATG; SLC9A3R1-AS1: TGTCCTCAGATGGTCAGCTC; SNRK-AS1: TTCAGTATCTCACTTCTGCC; MAL2-AS1 TACATATTCTCGCTTCACTC; SNHG11:GACTATCCAGGGACATCCTC; SMAD5-AS1:TGTGATTGTTCCTATACAGC, CATTATGAAATTAGTGTTCC; GEMIN7-AS1: ACAACTTGAAGATGGGATGC; CASC11: TTAGTGATGCCGAATTTCAT; USP3-AS1: CTTCTTCCTTGTTCTGTAGC; PYCARD-AS1: GTTCATGGTCTTTAATCTCC; LINC02035: TTACTACCTGACCTAACAGT; STARD4-AS1: TCACATTTGACAAGGAGCTC; DGUOK-AS1: TTATTTCGCACCCTCCATGC; LNCOC1: TGAGTCCGGAAAGCAGTCTT; VIM-AS1: AATTGTTCAGATGCCAAGGC, AAGAGGACCAGTGCCCATTC.

### 4.4 Cell migration assay

For the cell scratch assay, keratinocytes (four replicates per group) were seeded into 96-well plates. When the keratinocytes reached 50%–60% confluence, they were transfected with ASOs targeting these lncRNAs or an ASO control. After 24 h, the cells reached confluence and a single scratch was made with smart scratch (i-Omics Biotech Inc). The cells were washed with full medium to remove the detached cells. The cells were subsequently incubated with complete DermaLife® K Medium and photographed at 0, 12, and 24 h with a Nikon eclipse Ti2 inverted microscope at 100× magnification. The wound areas were measured using ImageJ software (National Institutes of Health, Bethesda, MD). The migration area was calculated using the following formula: Migration area (%) = (A0−An)/A0 × 100%, where A0 represents the initial wound area and An denotes the remaining wound area at the measurement point.

### 4.5 Cell proliferation assay

Cell proliferation was assessed using the CCK-8 assay (Vazyme, A311-01). Keratinocytes (six replicates per group) were seeded into 96-well plates with 100 μl medium in each well. Subsequently, when the keratinocytes reached 40%–50% confluence, they were transfected with ASOs targeting the lncRNAs or an ASO control. After 24 h, 10 μl of CCK-8 solution was added to each well and incubated in the dark for four hours. The absorbance at 450 nm was measured using a BioTek Synergy H1 Microplate Reader to determine cell survival rates. Cell survival/proliferation was quantified by absorbance of the test wells minus the optical density of the blank wells.

### 4.6 RNA extraction and qRT–PCR

Total RNA was extracted from cells using the RNAiso Plus reagent (9,109, Takara). PrimeScript™ RT Master Mix (Perfect Real Time) (RR036A, Takara) was used to reverse transcribe the total RNA (500 ng) to cDNA. In this study, real-time PCR was performed using the TB Green® Premix Ex Taq™ II (Tli RNaseH Plus) (RR820A, Takara). Amplification and detection of the lncRNAs were performed using a LightCycler 96 System (Roche, 05815916001) following the manufacturer's instructions. GAPDH was used as an internal control.

The primers used in this study were as follows: GAPDH Forward: GGTGTGAACCATGAGAAGTATGA, Reverse: GAGTCCTTCCACGATACCAAAG; KRT10 Forward: CGAAGGAGTTGGAGGTGTTT, Reverse: GCAGAGCTACCTCATTCTCATAC; LOR Forward: CGAAGGAGTTGGAGGTGTTT, Reverse: GGCTTCTTCCAGGTAGGTTAAG; FLG Forward: ATGAGCAGGCACGAAACA, Reverse: CCTGAGTGTCCAGACCTATCTA; IVL Forward: CTGTTCCTCCTCCAGTCAATAC, Reverse: CTTCTGCTGTTGCTCACATTC.

## 5 Statistics

The experiments were conducted with at least three replicates per group. The data were expressed as mean ± standard deviation (SD). Statistical significance was tested by Two-tailed Student's *t*-test for comparing two groups. Differences between groups were computed using two-way or one-way repeated-measures ANOVA. *P*-value <0.05 was considered statistically significant.

## Data Availability

Publicly available datasets were analyzed in this study. This data can be found here: https://www.ncbi.nlm.nih.gov/geo/query/acc.cgi?acc=GSE137897.
