# Supplementary material for: Single-cell profiling and functional screening reveal crucial roles for lncRNAs in the epidermal re-epithelialization of human acute wounds
Source: Front Surg. 2024 Feb 26;11:1349135. doi: 10.3389/fsurg.2024.1349135 (PMC10925684; doi:10.3389/fsurg.2024.1349135)
Supplement: Supplementary file 1 [file Table1.docx]

**Supplementary Material**


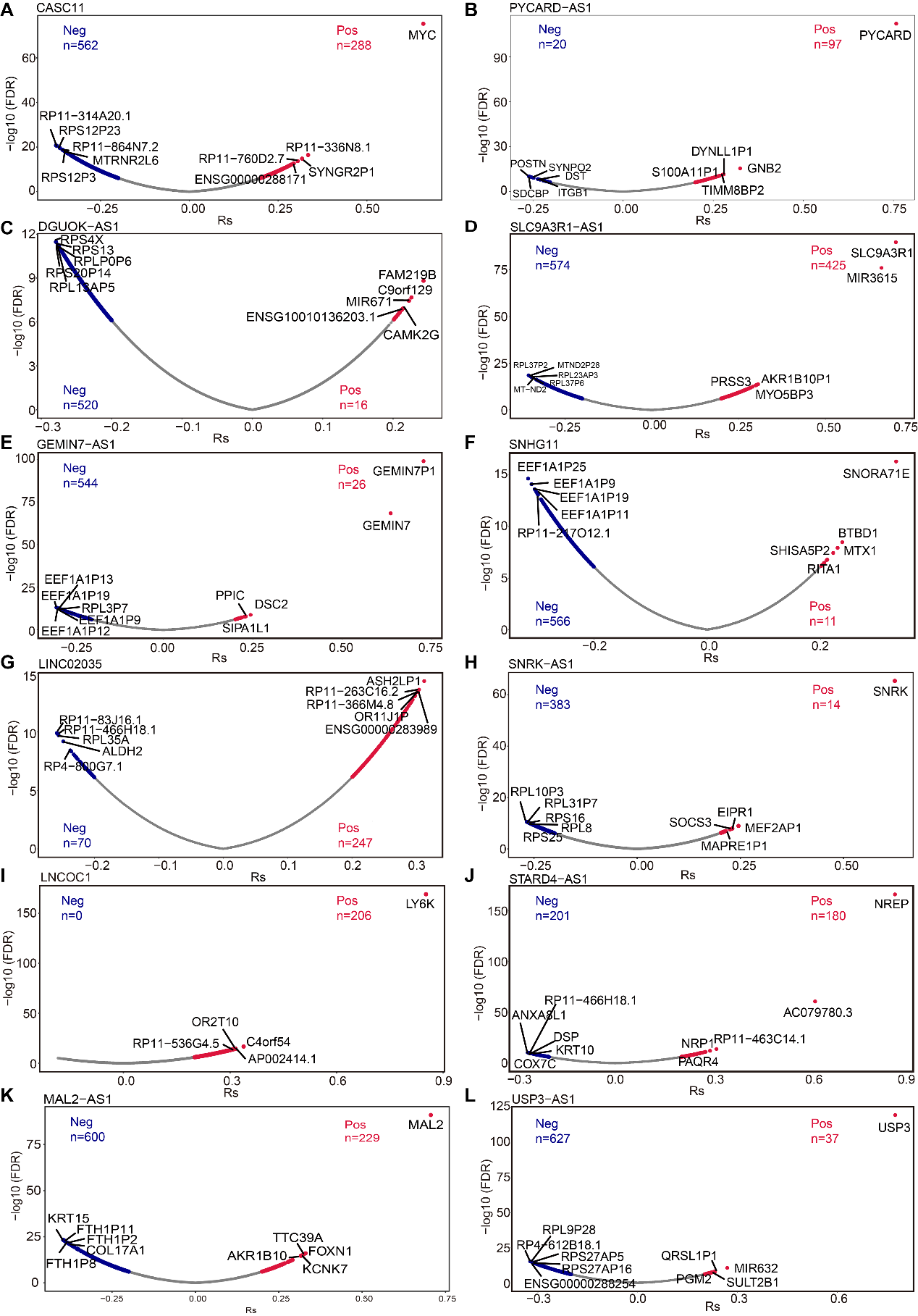


Figure S1. Correlation Analysis of lncRNA and Protein-Coding Genes. Volcano plot showed the mRNAs significantly associated with CASC11 (A), PYCARD-AS1(B), DGUOK-AS1(C), SLC9A3R1-AS1(D), GEMIN7-AS1(E), SNHG11(F), LINC02035(G), SNRK-AS1(H), LNCOC1(I), STARD4-AS1(J), MAL2-AS1(K), USP3-AS1(L) (FDR<0.05 and |RS|≥ 0.2). Positive correlation mRNAs were shown in the right (Pos). The negative correlation mRNAs were shown in the left (Neg).


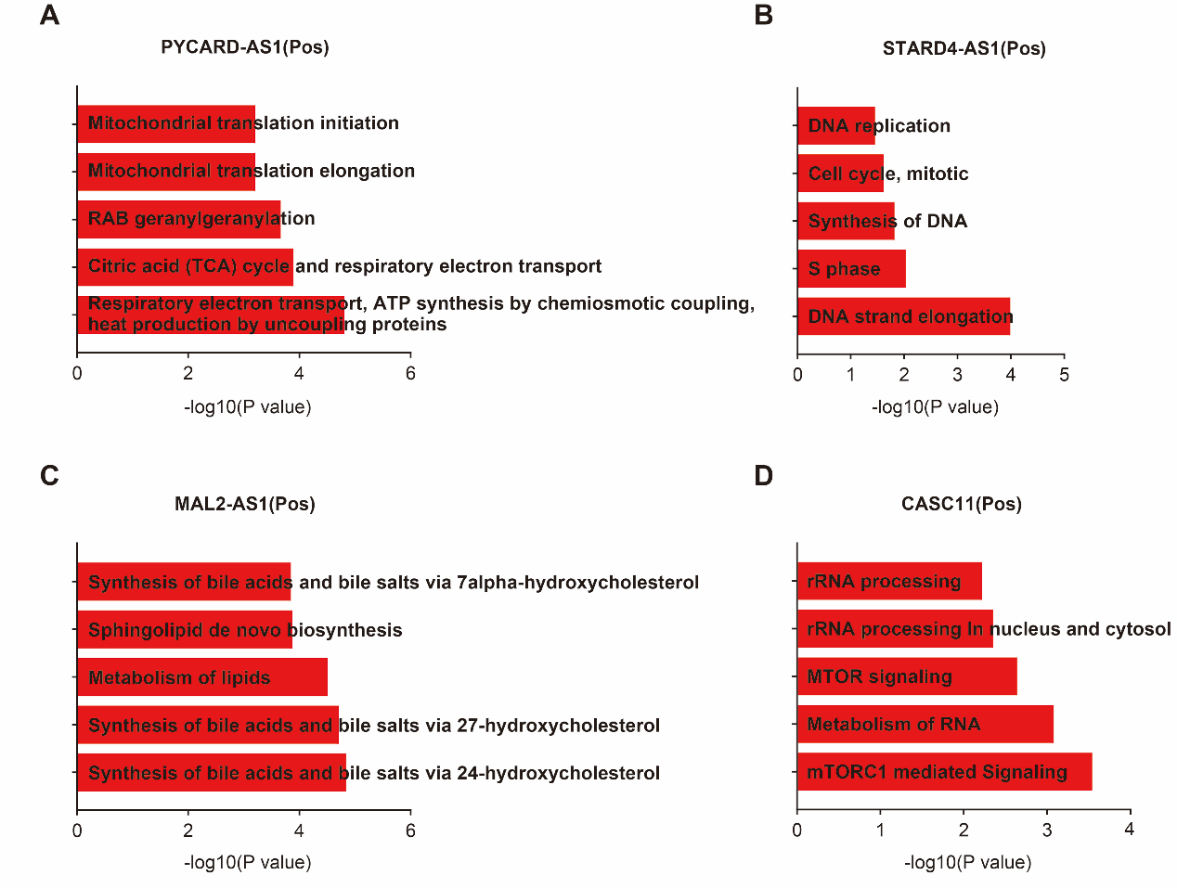


Figure S2. Correlation Analysis of lncRNA and Protein-Coding Genes. The Enrich R pathway analysis of the positive correlation coding genes with PYCARD-AS1 (A), STARD4-AS1(B), MAL2-AS1(C) and CASC11(D).

Tables S1.Top 15 lncRNAs conservation analysis (lncBook 2.0) (1)

| Gene Symbol | Zebrafish | Mouse | Homologous Gene（mouse） |
| --- | --- | --- | --- |
| *LINC02581* | Not conserved | Q90 | *Rnf227* |
| *VIM-AS1* | Q75 | Q50 | *Gm54102* |
| *PYCARD-AS1* | Not conserved | Q50 | *Gm45205* |
| *CASC11* | Not conserved | Q50 | *Gm37947* |
| *SLC9A3R1-AS1* | Not conserved | Q75 | *—* |
| *MAL2-AS1* | Not conserved | Not conserved | *—* |
| *SNHG11* | Not conserved | Q50 | *Snhg11* |
| *SNRK-AS1* | Q50 | Q90 | *—* |
| *STARD4-AS1* | Q75 | Q90 | *—* |
| *LINC02035* | Not conserved | Q75 | *—* |
| *DGUOK-AS1* | Not conserved | Q50 | *—* |
| *SMAD5-AS1* | Not conserved | Q75 | *—* |
| *LNCOC1* | Not conserved | Not conserved | *—* |
| *GEMIN7-AS1* | Not conserved | Q50 | *—* |
| *USP3-AS1* | Q50 | Q90 | *Gm15563* |

Four-degree categorization: Not conserved, Q50, Q75 and Q90

**Reference**

1. Li Z, Liu L, Feng C, Qin Y, Xiao J, Zhang Z, et al. LncBook 2.0: integrating human long non-coding RNAs with multi-omics annotations. Nucleic Acids Research. 2023 Jan 6;51(D1):D186–91.
